# RURALITY AND RACE IN WYOMING AND MISSISSIPPI: THE INTERSECTION OF PLACE AND RACE-BASED DISPARITIES

**DOI:** 10.1093/geroni/igae098.1823

**Published:** 2024-12-31

**Authors:** Taylor Jansen

**Affiliations:** University of Massachusetts Boston, Boston, Massachusetts, United States

## Abstract

Across the US, rural populations tend to be “older, poorer, and sicker” than their urban counterparts as they report higher morbidity, mortality, and greater risk of death due to unintentional injuries. In an already disadvantaged population, rural minorities tend to report even worse health outcomes. This study aims to examine the health disparities faced by rural populations by examining county-level 65+ outcomes in the predominately rural states of Wyoming (WY) and Mississippi (MS). Data from the 2023 WY Healthy Aging Data Report (HADR) and MS HADR was utilized to conduct bivariate statistical analyses and mapping to compare rural/frontier, and urban counties in WY and MS. Findings suggest rurality is protective of health in WY, where 21 of 23 counties are classified as rural or frontier, as these counties reported lower morbidity, despite lower access to primary care physicians (PCPs) [Rural: Mean(SD)=46.19(32.67); Urban: M(SD)=245(38.18)] and hospitals [Rural: Mean(SD)=1.1(0.44); Urban: M(SD)=2(1.41)]. Conversely, bivariate analyses found the two frontier counties with a Native American reservation reported the lowest life expectancy [M(SD)=77.7(1.7); WY: M(SD)=80.34(2.26)] and highest homicide rates [M(SD)=4.54(6.42); WY: M(SD)=0.83(2.31)] and COVID-19 mortality [M(SD)=476.64(52.43); WY: M(SD)=356.61(139.24)]. Similarly, predominately Black, rural counties in MS reported worse rates for 26 of 28 indicators of health and lowest access to care [PCPs: M(SD)= 18.52(20.56); Hospitals: M=1.06(0.65)] compared to rural, predominately White counties [PCPs: M(SD)= 23.88(19.92); Hospitals: M(SD)= 1.11(0.64)], and MS state averages [PCPs: M(SD)= 43.54(93.45); Hospitals: M(SD)= 1.23(1.10)]. These findings speak to the unique health disparities rural populations face at the intersection of place and race.

